# The Management of Intraoperative Spinal Cord Injury – A Scoping Review

**DOI:** 10.1177/21925682231196505

**Published:** 2024-03-25

**Authors:** Nader Hejrati, Nisaharan Srikandarajah, Mohammed Ali Alvi, Ayesha Quddusi, Lindsay A. Tetreault, James D. Guest, Rex A.W. Marco, Steven Kirshblum, Allan R. Martin, Samuel Strantzas, Paul M. Arnold, Saumyajit Basu, Nathan Evaniew, Brian K. Kwon, Andrea C. Skelly, Michael G. Fehlings

**Affiliations:** 1 Division of Neurosurgery, Krembil Neuroscience Centre, Toronto Western Hospital, 7989 University Health Network, Toronto, ON, Canada; 2 Department of Neurosurgery & Spine Center of Eastern Switzerland, Cantonal Hospital St.Gallen, St.Gallen, Switzerland; 3 Institute of Medical Science, 7938 University of Toronto, Toronto, ON, Canada; 4 Department of Neurology, NYU Langone Medical Center, New York, NY, USA; 5 Department of Neurosurgery and The Miami Project to Cure Paralysis, The Miller School of Medicine, 12235 University of Miami, Miami, FL, USA; 6 Department of Orthopedic Surgery, 570987 Houston Methodist Hospital, Houston, TX, USA; 7 Kessler Institute for Rehabilitation, 12286 Rutgers New Jersey Medical School, Newark, NJ, USA; 8 Department of Neurological Surgery, 8789 University of California Davis, Davis, CA, USA; 9 Division of Neurosurgery, Department of Surgery, The Hospital for Sick Children, 7938 University of Toronto, Toronto, ON, Canada; 10 Department of Neurosurgery, University of Illinois Champaign-Urbana, Urbana, IL, USA; 11 Kothari Medical Centre, Kolkata, India; 12 McCaig Institute for Bone and Joint Health, Department of Surgery, Orthopaedic Surgery, Cumming School of Medicine, 2129 University of Calgary, AB, Canada; 13 Department of Orthopaedics, 8166 University of British Columbia, Vancouver, BC, Canada; 14 International Collaboration on Repair Discoveries (ICORD), 8166 University of British Columbia, Vancouver, BC, Canada; 15 Aggregate Analytics, Inc., Fircrest, WA, USA; 16 Division of Neurosurgery and Spine Program, Department of Surgery, 7938 University of Toronto, Toronto, ON, Canada

**Keywords:** intraoperative spinal cord injury, checklists, care pathways, guidelines

## Abstract

**Study Design:**

Scoping Review.

**Objective:**

To review the literature and summarize information on checklists and algorithms for responding to intraoperative neuromonitoring (IONM) alerts and management of intraoperative spinal cord injuries (ISCIs).

**Methods:**

MEDLINE® was searched from inception through January 26, 2022 as were sources of grey literature. We attempted to obtain guidelines and/or consensus statements from the following sources: American Association of Neuromuscular & Electrodiagnostic Medicine (AANEM), American Academy of Neurology (AAN), American Clinical Neurophysiology Society, NASS (North American Spine Society), and other spine surgery organizations.

**Results:**

Of 16 studies reporting on management strategies for ISCIs, two were publications of consensus meetings which were conducted according to the Delphi method and eight were retrospective cohort studies. The remaining six studies were narrative reviews that proposed intraoperative checklists and management strategies for IONM alerts. Of note, 56% of included studies focused only on patients undergoing spinal deformity surgery. Intraoperative considerations and measures taken in the event of an ISCI are divided and reported in three categories of *i) Anesthesiologic, ii) Neurophysiological/Technical, and iii) Surgical management strategies.*

**Conclusion:**

There is a paucity of literature on comparative effectiveness and harms of management strategies in response to an IONM alert and possible ISCI. There is a pressing need to develop a standardized checklist and care pathway to avoid and minimize the risk of postoperative neurologic sequelae.

## Introduction

Though rare, intraoperative spinal cord injury (ISCI) is one of the most feared and serious complications of any spine surgery.^1,2^ ISCI can occur due to a variety of factors, including direct spinal cord trauma, ischemia, and traction during manipulation of the spine, for instance during deformity correction.^3^ The Stagnara wake-up test, which requires the reversal of general anesthesia and assessment of voluntary lower limb movements, and the ankle clonus test were previously the only methods of detecting ISCIs.^4,5^ Intraoperative neuromonitoring (IONM) is now widely used to provide continuous monitoring of spinal cord function during spine surgery. Individual monitoring setups have given way to multimodal IONM techniques that combine somatosensory-evoked potentials (SSEPs) and motor-evoked potentials (MEPs), as well as electromyography (EMG).^4,6,7^ This combined approach has the potential to improve the sensitivity and specificity of detecting neurological damage during spine surgery (although the precise combinations of modalities are variably reported in the literature).

The presence of significant deformity, cardiopulmonary comorbidities, extrinsic spinal cord compression and labile intraoperative mean arterial pressures are among the risk factors for IONM signal changes.^3,8^ The ability to detect and respond to these changes intraoperatively has resulted in a decrease in the rate of new or worsening neurologic deficits in this population, as the use of neuromonitoring may allow for rapid action to be taken to reverse the course of neurologic dysfunction.^9,10^ Although the ideal goal is to eliminate surgical complications, a more realistic goal is to consistently optimize responses to neuromonitoring changes so that permanent deficits occur as infrequently as possible^11^ While several algorithms for responding to changes in neuromonitoring have been developed, none have been widely accepted or consistently used in general practice for a variety of reasons^10,12^ Nonetheless, there is evidence that checklists improve care in other areas of surgical intervention. Evidence suggests that surgeon performance suffers under stress and time constraints, and that checklists are useful tools in these situations^13,14^ According to previous studies, using a cognitive aid, such as a checklist, correlates with better management of operating room crises. A recent study evaluating the use of checklists in crisis situations in the operating room found that using a checklist resulted in a six-fold reduction in failure to adhere to critical management steps^15^ A checklist may systematically order parameters that could reverse the detected warning signal.

As discussed, there are a number of checklists that have been published in the past. In the current manuscript, we have summarized the management strategies that have been previously proposed in response to an IONM alert.

## Materials and Methods

This manuscript intends to summarize information on checklists and algorithms for responding to IONM alerts and is part of conceptual questions from a proposed systematic review on ISCI management and risk factors for ISCI (registered protocol: PROSPERO CRD42022298841). The intended contextual and key questions, as well as PICOTS scope, from this protocol are published separately within this focus issue. Addressing the contextual questions from the original protocol was based on the U.S. Preventive Services Task Force methods^16^ for contextual questions and based on citations identified via the scoping search of peer reviewed and gray literature done based on the original registered systematic review protocol.

### Literature Search Strategies

Literature Databases: MEDLINE® was searched from inception through January 26, 2022, as were sources of gray literature, based on the original protocol and limited targeted searches for guidelines were done. Citations suggested by the authors and guideline development group were considered. We attempted to obtain guidelines and/or consensus statements from the following sources: American Association of Neuromuscular & Electrodiagnostic Medicine (AANEM), American Academy of Neurology (AAN), American Clinical Neurophysiology Society, NASS (North American Spine Society), and other spine surgery organizations.

### Criteria for Inclusion/Exclusion of Studies

General inclusion and exclusion criteria regarding patient populations and interventions for the original contextual and key questions were used to select citations describing IONM checklists, pathways for addressing alerts, and clinical guidance for managing ISCI.

## Results

### Study Selection

We searched the literature and identified 16 checklists or treatment algorithms that provided recommendations for the management of, and response to, an IONM warning/alert during spinal surgery. Only publications that provided a formal checklist or treatment algorithms were included. Most checklists and algorithms were developed by professional societies or for hospital-specific protocols. The checklists and algorithms identified have been summarized.

### Characteristics of Included Studies

Of the 16 studies reporting on management strategies for ISCIs, two were publications of consensus meetings which were conducted according to the Delphi method,^17,18^ and eight were retrospective cohort studies.^3,19-25^ The remaining six studies were narrative reviews that propose intraoperative checklists and management strategies for IONM alerts.^11,26-30^ Of note, 56% of included studies had a sole focus on patients undergoing spinal deformity surgery.^3,11,17-21,23,25^

Intraoperative considerations and measures taken in the event of an ISCI are herein reported and divided into three categories of *i) Anesthesiologic, ii) Neurophysiological/Technical, and iii) Surgical management strategies* (Tables 1–3).

**Table 1.** Management Strategies for Intraoperative Spinal Cord Injury – Anesthetic Systemic Considerations.

Author (Year), design, field of spine surgery	MANAGEMENT STRATEGIES						
	Anesthetic systemic management						
	Blood pressure	Anesthetics	Body temperature	Blood volume and hematocrit	Blood gases	Wake up test	Other strategies
Vitale, (2014) Delphi consensus meeting, deformity	- Optimize MAP	- Revisit anesthetic considerations and confirm that they are optimized	- Establish normothermia	- Optimize hematocrit	- Optimize blood pH and pCO_2_	*-* Perform wake-up test	- NA
Ziewacz, (2012) Narrative review, general	- Increase MAP to 90-100 mmHg, if no changes consider increase to >100 mmHg	- Check if neuromuscular blockade (muscle relaxant) given	- Check temperature	- Check hemoglobin/hematocrit and aim for hemoglobin >9-10 g/L	- NA	- Consider wake-up test	- NA
		- Assess anesthetic depth (BP, RR, HR, BIS monitor)					
		- Lighten depth of anesthesia (reduce MAC, temporarily eliminate inhaled agents, reduce intravenous anesthetics, add adjuvant agents, eg ketamine)					
Buhl, (2021) Analytical Narrative review, general	- Increase perfusion pressure	- Eliminate volatile anesthetics	- Establish normothermia	- Consider blood transfusion	- Establish normocarbia	- Consider wake-up test	- Establish normoglycemia
		- Decrease anesthetic depth					
		- Consider ketamine to enhance IONM signals			- Increase FiO_2_		- Correct electrolyte abnormalities
Bible, (2015) Instructional course Lecture, narrative review, cervical	- Establish MAP >85 mmHg	- Different anesthesia agent or bolus	- Temperature >36.5°C	- Consider transfusion with hemoglobin goal of >8 g/L	- NA	- Consider wake-up test	- NA
Pahys, (2009) narrative review, idiopathic scoliosis	- Aim for MAP >80 mmHg	- NA	- Check temperature >36.5°C	- Check hemoglobin level	- Optimize oxygenation	- Perform wake-up test	- Check glucose levels
Jain, (2015) Mini review, general	- Elevate MAP to 80-90 mmHg	- Align anesthesia with surgical plans (eg anesthetic gas, nitrous oxide)	- Reversal of hypothermia	- Reverse any anemia <8 g/L	- Reverse hypocapnic alkalosis	- Consider Stagnara wake-up test	- NA
					- Increase patient oxygenation		
Tsirikos, (2019) retrospective cohort, deformity	- Elevate MAP >80 mmHg	- Reverse muscle relaxation	- Raise temperature >36.5°C	- NA	- NA	- NA	- NA
		- Change anesthetic agent					
Jarvis, (2013) retrospective cohort, deformity	- Elevate MAP >70 mmHg	- Rule out anesthesia-related neurophysiological changes	- NA	- NA	- Optimize oxygenation	- NA	- NA
Acharya, (2017) retrospective cohort, deformity	- Increase MAP >90 mmHg, if no improvements consider increase to >100 mmHg	- Check if bolus of anesthesia drug or muscle relaxants given	- Increase core temperature	- Check hemoglobin	- Check oxygenation	- Perform a Stagnara wake-up test	- NA
		- Lighten depth of anesthesia (stop inhalational agents, reduce IV anesthetics, add adjuvant eg ketamine)					
Sahinovic, (2021) narrative review, general	- Optimize blood pressures	- Optimize anesthetic depth	- Ensure normothermia	- Optimize hematocrit	- Optimize oxygenation	- Perform a wake-up test	- NA
		- Ensure no neuromuscular blocking agent given					
		- Consider hypnotic drug rotation					
		- Consider adding adjuvant drugs (eg Ketamine)		- Optimize blood volume	- Optimize ventilation		
Lenke, (2022) Delphi consensus meeting, deformity	- Elevate MAP	- Review possible anesthetic changes	- Normalize temperature	- Reverse anemia	- Normalize blood pH and pCO_2_	- Consider wake-up test	- NA

Note. BIS: bispectral index; BP: blood pressure; HR: heart rate; IONM: intraoperative neuromonitoring; MAP: mean arterial pressure; RR: respiratory rate.

**Table 2.** Management Strategies for Intraoperative Spinal Cord Injury – Neurophysiological/Technical and Other Considerations.

Author (Year), design, field of spine surgery	Management Strategies						
	Neurophysiological & technical management				Other management strategies		
	Electrode set-up	Stimulation parameters	Patient positioning	Repeat testing of evoked potentials	Corticosteroids	Peer consultation	Other pharmacotherapies
Vitale, (2014) Delphi consensus meeting, deformity	- Check electrodes and connections	- NA	- Check neck and limb positioning; check limb position on table especially if unilateral loss	- NA	- Consider IV steroids: MPS 30 mg/kg in first hour, then 5.4 mg/kg/hour for the following 23 hours	- Consider consulting with a colleague	- NA
Ziewacz, (2012) Narrative review, general spine	- Check leads and rule out pull-out	- NA	- Check extremity position in case of plexus palsy	- Repeat trials of MEPs and SSEPs to rule out potential false positive	- Consider steroid administration	- NA	- Consider topical or IV calcium channel blocker
	- Consider adding leads in proximal muscle groups if possible						
Buhl, (2021) Analytical, Narrative Review, general spine	- Tighten loose electrode connections	- Increase stimulation intensity	- Look for external compression from inadvertent tourniquets or malpositioning	- NA	- NA	- NA	- NA
	- Reposition/replace electrodes						
	- Check electrode impedances	- Change priming pulses	- Refrain from leaning on patient				
Bible, (2015) Instructional Course Lecture, Narrative review, cervical	- Reconnect/Reposition electrodes	- NA	- Check neck position for hyperextension and reposition if necessary	- NA	- Consider IV steroids	- NA	- NA
			- Untape shoulders, check elbows				
Pahys, (2009) Narrative review, idiopathic scoliosis	- NA	- NA	- NA	- NA	- Consider IV steroids: MPS bolus of 30 mg/kg over 15 min, then 5.4 mg/kg/hour as a 23-hour infusion	- NA	- NA
Jain, (2015) Mini review, general spine	- Check for electrode dislodgement	- NA	- NA	- NA	- NA	- NA	- NA
Tsirikos, (2019) Retrospective cohort, deformity	- Exclude dislodgement of electrodes	- Adjust stimulation parameters	- Change patient positioning	- Repeat MEPs at 5-min intervals for 30 minutes	- NA	- NA	- NA
Jarvis, (2013) Retrospective cohort, deformity	- NA	- NA	- NA	- NA	- Consider steroid administration: IV dexamethasone	- NA	- NA
Acharya, (2017) Retrospective cohort, deformity	- Check electrodes	- Increase stimulus strength	- Check limb position	- Repeat trials of MEP	- Consider steroid administration	- NA	- NA
	- Reduce electrical interference						
Sahinovic, (2021) Narrative review, general	- Check electrodes and connections	- Consider increasing stimulation intensity	- NA	- Repeat MEP measurements	- NA	- Consider consulting with a colleague	- Consider topical or IV calcium channel blocker
	- Check for electrical interference						
Lenke, (2022) Delphi consensus meeting, deformity	- NA	- NA	- Check and adjust patient positioning (ensure appropriate bolster placement; consider spinal cord impingement due to prone positioning or spinal instability)	- NA	- Consider IV steroids	- Always consider consultation with a colleague prior to proceeding	- NA
Lee, (2006) Retrospective cohort	- NA	- NA	- Reverse neck extension	- NA	- NA	- NA	- NA
Lewis, (2011) Retrospective cohort	- Rule out equipment and technical causes	- NA	- NA	- Perform frequent MEP’s	- NA	- NA	- NA
Vitale, (2010) Retrospective cohort	- Check technical problem and correct, eg leads positioning	- NA	- NA	- NA	- NA	- NA	- NA
	- Check for interference						
Rajappa, (2021) Retrospective cohort	- Rule out technical glitches with the recording system or owing to dislodgement of electrodes	- NA	- NA	- Repeat potentials after few minutes	- NA	- NA	- NA
Ferguson, (2014) Retrospective cohort	- Check all connections	- NA	- Check all limbs	- NA	- Consider steroid therapy	- NA	- NA

Note. IV: intravenous; MEPs: motor evoked potentials; MPS: methylprednisolone; SSEPs: somatosensory evoked potentials.

**Table 3.** Management Strategies for Intraoperative Spinal Cord Injury – Surgical Considerations.

Author (Year), design, field of spine surgery	Management Strategies				
	Surgical management				
	Traction and deformity correction	Hardware malposition and compressive forces	Interruption or staging of procedure	Team communication	Local irrigation
Vitale, (2014) Delphi consensus meeting, deformity	- Remove traction	- Remove screws and probe for breach	- Intraoperative pause: Stop case and announce to the room	- Discuss events and actions just prior to signal loss	- NA
	- Decrease/remove distraction or other corrective forces	- Evaluate for spinal cord compression, examine osteotomy and laminotomy sites		- Eliminate extraneous stimuli (eg music, conversations, etc.)	
	- Remove rods	- Intraoperative and/or perioperative imaging (eg O-arm, fluoroscopy, x-ray) to evaluate implant placement	- Consider continuing surgical procedure vs staging procedure	- Summon attending anesthesiologist, senior neurologist or neurophysiologist, and experienced nurse	
Ziewacz, (2012) Narrative review, general spine	- Consider reversing correction of a spinal deformity	- Assess field for structural cord compression (misplaced hardware or bone graft, osteophytes, or hematoma)	- Stop current manipulation	- Communicate key findings and relevant actions to each other	- NA
		- Perform further decompression if stenosis is present	- Consider aborting surgery		
Buhl, (2021) Analytical, narrative review, general spine	- Release distraction rods	- Eliminate pressure points in the surgical field (adjust retractors)	- Consider aborting the procedure	- Communication between surgeon, neuromonitorist, and anesthesiologist	- NA
		- Inspect hardware (adjust position of hardware and screws)			
Bible, (2015) Instructional course Lecture, narrative review, cervical	- Lessen distraction/correction	- Remove compressive element	- Consider aborting the procedure	- NA	- NA
		- Radiographically check placement of instrumentation, cage, graft and remove if necessary			
		- Emergent MRI to rule out compression			
Pahys, (2009) Narrative review, idiopathic scoliosis	- Remove correction	- Remove implants (unless spine is unstable)	- NA	- NA	- NA
	- Consider modest correction or in situ fusion				
Jain, (2015) Mini review, general spine	- Reverse distraction	- Widen decompression	- NA	- Communicate with the anesthesia team to ensure anesthesia and surgical plans are well aligned	- NA
Tsirikos, (2019) Retrospective cohort, deformity	- Remove instrumentation and reverse surgical manoeuvres back to last normal MEPs	- Remove instrumentation and reverse surgical manoeuvres back to last normal MEPs	- Halt surgery	- Close cooperation between the surgical, anesthetic, and neurophysiology team	- NA
			- Consider abandoning procedure		
			- Consider completion of surgery in a staged manner		
Jarvis, (2013) Retrospective cohort, deformity	- Remove traction	- Reopening of osteotomy and ensure no bone is pressing on the spinal cord, manipulation and cage adjustment, and reclosure of the osteotomy	- Cessation of any further surgical intervention	- NA	- NA
	- Reduce traction weights				
	- Insert stabilizing rod				
	- Close osteotomy				
Acharya, (2017) Retrospective cohort, deformity	- Release traction	- Check dura for compression	- Stop current manipulation	- Reduce noise	- Perform warm saline wound wash
	- Reverse corrective maneuver & rod removal				
	- Confirm stability of spine	- Look for implant malposition	- Consider aborting surgery		
Sahinovic, (2021) Narrative review, general	- Remove rods	- Remove screw	- Consider staging/abandoning the surgical procedure	- Clearly communicate the problem to the whole team	- Irrigate spinal cord with saline
	- Stop traction	- Check dura for compression			
Lenke, (2022) Delphi consensus meeting, deformity	- Remove/reduce traction unless necessary for spinal stability	- Assess for dural compression circumferentially, intraoperative imaging (x-ray, fluoroscopy, CT, US)	- Consider aborting surgery and return at a later date	- Minimize distraction and gain attention of the operating room	- NA
		- Evaluate screws (palpation, x-ray, intraoperative CT) and remove as indicated			
	- Stabilize with temporary rod(s) fixation	- Assess for dural compression/tension (eg possible pedicle fracture impinging on neural elements) and decompress as indicated (eg laminectomy, apical pediculectomy)			
Lee, (2006) Retrospective cohort	- Reverse any antecedent surgical event, eg distraction	- NA	- NA	- NA	- NA
Lewis, (2011) Retrospective cohort	- Remove traction and remove rods	- Rule out misplaced implants	- Terminate surgery and awaken patient	- NA	- NA
		- Conduct appropriate cross-sectional image of spine if no improvement			
Vitale, (2010) Retrospective cohort	- Check mechanical event and take action, eg relax curve correction	- Check mechanical event and take action	- NA	- NA	- NA
Rajappa, (2021), Retrospective cohort	- Reverse surgical procedure to last normal evoked potentials and consider moderate correction or modification of the plan	- Remove any instrumentation placed	- Surgeon alerted and halt on operation	- Surgeon to be alerted	- Perform warm saline irrigation of surgical site
Ferguson, (2014), Retrospective cohort	- Reverse most recent action, release correction/rod	- Investigate for breach, neural compression	- Consider aborting procedure	- NA	- NA
		- Remove most recent instrumentation, explore screw tract, palpate osteotomy/exposed areas for bone, hematoma, instrument, hemostatic agent compressing cord			
		- Obtain MRI/CT if motor function impaired after wake-up test			

Note. CT: computed tomography; MEPs: motor evoked potentials; SSEPs: somatosensory evoked potentials; VCR: vertebral column resection; US: ultrasound.

### Anesthesiologic Considerations

#### Anesthetics

A majority of included studies (14/16) acknowledged that the modalities of the anesthetic regimen might confound the interpretation of neuromonitoring changes and therefore warrant critical review. As such, suggested actions to be considered ranged from adjusting the anesthetic depth (eg as indicated by the blood pressure, respiratory rate, heart rate, or bispectral index monitoring),^21,27,30^ to specific pharmacological recommendations, such as using different/adjuvant anesthetic agents^26,30^ and making sure that muscle relaxants or inhalational anesthetics are metabolized or stopped.^19,24,29^

#### Blood Pressure

While all included studies uniformly suggested optimization of blood pressures if intraoperative loss of monitoring signals were suspected to be related to impaired spinal cord perfusion, there was some degree of heterogeneity with regard to the blood pressure targets. While the majority of studies suggested MAP values above 80 mmHg,^11,19,21,22,24-27,29^ two studies propose MAP targets of ≥70 mmHg.^20,23^ In fact, out of the nine studies that recommended MAP targets of at least 80 mmHg, two studies proposed targets of >85 mmHg,^22,26^ and two studies suggested MAP targets of 90-100 mmHg and >100 mmHg if no improvements with lower thresholds were observed.^21,27^ Five out of the 16 included studies suggested optimizing blood pressures without further specifying pressure targets.^3,17,18,28,30^

#### Body Temperature

Most studies suggested checking for core temperature and increasing the body temperature if hypothermia is noticed.^11,17-19,21,24-30^ Four studies proposed a core temperature of at least 36.5° in order to exclude any thermic effects on changes of intraoperative evoked potential signals.^11,19,24,30^ Some of the described measures to increase core temperature included an increase in room temperature, the use of warming blankets, or the application of warm wound irrigation.^26^

#### Blood Volume, Hematocrit, Blood Gases and Other Management Strategies

Anemia and, as a consequence, impaired tissue oxygenation was recognized as an important contributor to loss of IONM signals and as such its correction was recommended by the majority of included studies.^11,17,18,21,25-30^ Hemoglobin values of >8 g/L^26,29^ or 9-10 g/L^27^ and a hematocrit of >30%^30^ were some of the targets proposed in order to address anemia-related loss of signals.

It is understood that neuronal tissues have higher levels of metabolic demands and therefore require adequate oxygenation for appropriate functioning. Therefore, optimizing blood pH and achieving normocapnia (eg through management of ventilation)^17,18,28-30^ as well as optimizing oxygenation (eg by fraction of inspired O2, FiO2)^11,20,21,23,28-30^ were recommended to achieve this goal.

Other systemic factors that potentially contribute to intraoperative signal changes include blood glucose levels and electrolyte derangements, and hence normalization of these parameters was suggested by Buhl et al and Pahys et al as part of the systemic management of ISCI.^11,28^

#### Wake up Test

If anesthetic, neurophysiologic/technical and surgical measures have not improved intraoperative signal loss, a wake-up test was considered as a last resort option by most of the included studies, when feasible.^3,11,17,18,21,22,24-30^

### Neurophysiological & Technical Considerations

#### Electrode Set-Up

Among the most common issues related to technical failures in the process of IONM is the disconnection of either stimulating or recording electrodes. Reconnecting or repositioning loosened electrodes was therefore suggested as an initial step in the management of potential technical failure.^3,17,19,21,23-30^ Technical optimization was further suggested through reduction of electrical interference from digital equipment such as warming blankets, electrocautery equipment, or magnetic devices.^3,21,29,30^ An example of how electrical interference can be ruled out was proposed in the use of free-running EMG’s.^30^

#### Stimulation Parameters

Adjustment of stimulation parameters may be needed, for example, if the use of inhalational anesthetics dampens the SSEP and/or transcranial MEP amplitudes.^19,21,28,30^ In such a scenario, increasing the stimulation intensity,^21,28,30^ using multipulse stimulations,^30^ or adapting other aspects of stimulation parameters (eg pulse width, interstimulus intervals, etc.)^28^ were among the factors suggested to optimize the configuration of stimulation parameters.

#### Repeat Testing of Evoked Potentials

It is understood that the detection of ISCI is a time-sensitive matter. Therefore, several studies suggested repeating evoked potential measurements and shortening assessment intervals in order to capture possible improvements of IONM signals after implementation of corrective measures and to rule out false positive signal changes in a timely manner.^19,21,23,24,27,30^ Some studies suggested time intervals of a few minutes^24^ while others porposed 5-minute intervals for 30 minutes.^19^

#### Patient Positioning

Cervical hyperextension, external pressure-related peripheral nerve injuries and traction of the brachial plexus are potential causes of patient positioning-related spinal cord or peripheral nerve injuries. Therefore, verifying proper patient positioning and correcting neck and limb position in the event of an ISCI was a key strategy by a great proportion of the included studies.^17-19,21,22,25-28^ The use of appropriate taping techniques (eg the shoulders in cervical spine surgery) and cushioning of extremities were some of the suggested measures to prevent positioning- and traction-related nerve damage.^26^ Other specific measures included refraining from leaning on the patient as this also might harbor risk of pressure-related nerve injury.^28^

### Surgical Considerations

#### Traction and Deformity Correction

If the occurrence of an ISCI is associated with an intraoperative deformity correction maneuver (ie during distraction), then there is uniform agreement that distractive forces on the spinal cord need to be reversed.^3,11,17-30^ Measures to reduce the traction on the spinal cord and thereby reduce spinal cord hypoperfusion included removal of the distraction rods or closing an osteotomy site.

However, if spinal instability, in particular following three-column osteotomies (eg vertebral column resection), is suspected to be the underlying cause of intraoperative signal changes, then the insertion of a temporary, stabilizing rod was suggested.^18,20,21^

#### Hardware Malposition and Compressive Forces

Mechanical compression of neural structures was recognized as a potential cause of intraoperative signal loss by most of the included studies.^11,17-21,26-30^ As such, ruling out hardware malposition and other compressive forces on the spinal cord and/or nerve roots was widely recommended. Measures to reverse or reduce compression on neural elements included adjusting or repositioning implants (such as interbody cages, grafts and screws),^11,17-21,26-28,30^ opening up the osteotomy site,^29^ widening the decompression and looking for compressive osteophytes, hematoma, or hemostatic agents.^25-27,29^ Finally, if no structural cause of intraoperative signal changes can be identified after surgical exploration, intra- and postoperative advanced imaging was suggested to assess for hardware malposition and cord compression.^11,18,23,25,26^

#### Interruption or Staging of the Procedure

If an intraoperative loss of signals is noticed, a temporary pause with the immediate cessation of any surgical manipulations along with a clear announcement to the operating room was suggested.^17,19-21,24,27,30^

If an underlying cause for IONM signal losses cannot be identified or the signal changes have not recovered after all the appropriate measures have been considered, then either aborting the intervention^18,19,21,23,25-28,30^ or a staged procedure were suggested.^17-19,30^

#### Team Communication and Peer Consultation

In the event of an IONM event, the importance of communication was emphasized between members of the team including the surgeon, neuromonitoring technician, anesthesiologist and nursing staff.^17-19,21,24,27-30^ Specific communication points included discussing events prior to signal loss and considering reversing actions^17,18^ as well as ensuring that anesthetic and surgical plans were aligned.^29^ Additional measures suggested to retain or gain control of the operating room in the critical setting of an IONM signal loss included elimination of extraneous stimuli (eg music, conversations, etc.) and reduction of noise.^17,18,21^

Considering a consultation with a colleague following an IONM alert was suggested by three studies.^17,18,30^ This would be preferably performed with a colleague experienced in the type of surgery in which a critical IONM event occurs and may be applicable at multiple stages perioperatively.^18^

#### Local Irrigation and Pharmacological Therapies

The three studies by Acharya et al, Rajappa et al and Sahinovic et al. suggested considering washing the wound with warm saline^21,24^ and irrigating the spinal cord with saline, respectively, in the context of an ISCI.^30^

Two studies (Ziewacz et al and Acharya et al) proposed considering topical or IV administration of calcium channel blockers.^21,27^ A number of studies (n = 8) considered the administration of steroids if earlier attempts of reversing intraoperative signal loss failed.^11,17,18,20,21,25-27^ The therapeutic regimens described included the administration of intravenous (IV) methylprednisolone 30 mg/kg for the first hour followed by 5.4 mg/kg for the following 23 hours, in keeping with the NASCIS II protocol for acute traumatic SCI.^11,17,18^

## Discussion

Based on the current evidence and summary of the available literature, management strategies after detection of intra-operative signal loss can be broadly categorized into three key areas; (i) Anesthetic/Systemic considerations, (ii) Neurophysiologic and Technical considerations, and (iii) Surgical strategies.

When first aware of an intraoperative signal loss, a general initial step in the management of a potential ISCI is to pause the surgery and communicate the possible adverse event to all team members present in the operating room. Stressful events may adversely affect human cognition, which is why checklists are required for care pathways that may be considered common sense during routine events.^31^ Checklists have been routinely used in high stress and time critical sectors such as the aviation industry. Various checklists which have been modeled on the basis of aviation checklists, such as the WHO surgical safety checklist, or the SURPASS (SURgical Patient Safety System) checklist have successfully resulted in a decrease in both complications and mortality rates.^32,33^ Similarly, the use of pre-defined and standardized treatment/assessment algorithms such as the basic life support (BLS) and the advanced cardiovascular life support (ACLS) guidelines allow healthcare providers to perform critical and effective actions in a timely manner, which in turn improves patient outcomes.^34^ Unequivocal communication within the patient care team, which generally consists of the anesthesiologist, neurophysiologist, nursing staff and the surgeon, is pivotal in order to maintain a high level of patient safety, especially in the setting of high risk procedures and events.^35^

An ISCI can occur during any phase perioperatively, even prior to skin incision. In such a scenario, one potential cause includes hyperextension of the cervical spine during intubation or patient positioning. An observation from the American Society of Anesthesiologists Closed Claims database showed that 57% of ISCI to the cervical spinal cord were found in patients with cervical spondylosis and/or disc herniations, as compared to 24% of ISCI that occurred in patients with cervical instability.^26,36^ Consequently, fiberoptic intubation instead of laryngoscopic intubation should be strongly considered in patients with preexisting degenerative changes of the cervical spine. Communication between the surgeon and the anesthesiologist prior to surgery is imperative in order to take appropriate precautions (eg avoidance of hyperextension during patient positioning) and to avoid iatrogenic SCI. Peripheral nerve and brachial plexus injury are other feared complications associated with improper patient positioning, which can have tremendous consequences for patients due to the loss of upper extremity function. Ulnar neuropathy, for example, is the commonest type of perioperative peripheral nerve injury encountered during cervical spine surgery with estimates of 28% of all claims for anesthesia-related nerve injury.^37^ Brachial plexus injuries, on the other hand, result from nerve stretching or compression with resulting ischemia of the vasa nervorum and commonly occur due to taping of shoulders during cervical spine procedures. The addition of neuromuscular blocking agents (NBA) further adds to the laxity of the shoulder, thereby allowing inadvertent excessive tractive forces on the brachial plexus. Adequate wrapping of the arms (eg with foam or gel pads), correct taping of shoulders and if a peripheral nerve injury is suspected intraoperatively, repositioning of extremities is strongly recommended. Finally, femoral artery compression during prone positioning can also lead to IONM loss. Palpation of pedal pulses and great toe pulse oximetry can detect this potential cause of IONM loss when no other technical issues are identified.^38,39^

Once an ISCI has occurred and potential technical sources of false-positive alarms have been ruled out (such as disconnection of electrodes or interference with technical devices), it is important to rule out anesthesiologic contributors to false-positive signal changes. As an example, while Propofol is an ideal hypnotic drug for the maintenance of anesthesia during spinal surgery with IONM, its hyperpolarizing effect mediated through gamma-aminobutyric acid synapses produces synaptic inhibition and may therefore dampen amplitudes of MEPs and SSEPs, as well as increase the latency of SSEPs. However, when administered in clinically relevant doses, its interference with IONM is negligible.^40^ As a consequence, it is important to optimize doses of administered hypnotic drugs while monitoring the anesthetic depth (eg with the use of target-controlled infusions and EEG-based monitors of anesthetic depth) and avoiding boluses, particularly during critical surgical maneuvers. Additionally, intraoperative administration of opioids synergistically enhances the hypnotic drug effect, thereby allowing for a dose reduction of hypnotic drugs and mitigating the influence of hypnotic drugs on IONM signals. Furthermore, opioids dampen adrenergic responses to surgical stimuli, which aids in maintaining hemodynamic stability and thus spinal cord perfusion.^30^ Inhalational anesthetics on the other hand have significant dose-dependent amplitude-lowering effects on MEPs and SSEPs, and are therefore not suitable during surgeries where IONM is used.^41^ Adjustment of stimulation parameters, such as increasing stimulation intensities or employing multipulse stimulations, can partially overcome the suppressive effects of volatile anesthetics, but these effects are limited with higher mean alveolar concentrations.^30,41^ NBA’s play an important role during spinal surgery, particularly during the initial phases of surgical exposure. However, they directly reduce MEP amplitudes in a dose-dependent manner and hence their administration is not recommended where MEP monitoring is employed. It is therefore important to align the timing of administration of NBA’s with the phases of surgical procedure (eg during anesthesia induction or surgical exposure).

Intraoperative spinal cord injuries are a time-sensitive matter and it is therefore crucial to immediately ensure adequate spinal cord homeostasis once an injury is suspected. Neuronal tissues have high metabolic demands, thus adequate spinal cord perfusion in conjunction with optimal blood oxygenation are the main pillars ensuring uninterrupted tissue oxygenation. An interplay between the surgeon and the anesthesiologist is warranted to optimize spinal cord oxygenation (eg through parameters of ventilation, inspiratory O2 fraction, hemoglobin and blood pressure). While specific target values remain elusive in patients with a suspected ISCI, normovolemia, normoxaemia^11,20,21,23,28-30^ and normobcarbia^17,18,28,29^ are among the factors that need to be maintained while any anemia should be ideally reversed.^11,17,18,21,25-30^ In analogy to the management of cerebral perfusion pressures in the context of traumatic brain injury, the MAP constitutes the driving force behind spinal cord perfusion. While the realm of ISCI research has not provided evidence to support specific MAP targets, a number of studies suggest MAP targets that are analogous to those seen in the field of traumatic SCI.^42^ A MAP target of >80 mmHg followed by a stepwise increase of up to 100 mmHg,^11,19,21,22,24-27,29^ if earlier thresholds have not reversed intraoperative signal changes, are among the most commonly chosen strategies. Clearly, prospective studies are warranted to strengthen the evidence and provide stronger recommendations with regard to optimal MAP targets.

Body core temperature directly influences signal changes of evoked potentials. Preclinical studies have demonstrated decreased central conduction velocity, and as a result increased latency and decreased amplitude of MEPs and SSEPs, under hypothermic conditions.^43,44^ As a consequence, animal studies have shown increased rates of false-negative SSEPs under hypothermia, rendering them a less reliable tool in predicting an adverse outcome.^43^ A clinical study of 90 patients, where latencies and amplitudes of SSEPS during different stages of scoliosis surgery were compared found that in 12 patients a 50% drop in SSEP amplitude was noted after exposure of the spine and before instrumentation and deformity correction.^45^ In two patients, amplitudes dropped beyond 50% but reversed following irrigation of the spine with warm saline, suggesting an association between neurophysiological changes and a decrease of local temperature. In order to minimize the rates of false-positive events, the authors propose the use of SSEP baselines obtained after spinal exposure instead of baselines obtained prior to skin incision.^45^ Increasing core temperature to normal ranges has converse effects and reduces EP latency in all modalities thereby reducing the rate of false alarms.^30,44^

While the great majority of included studies recommend reversing any potential hypothermia and establishing normothermia, it is pivotal to avoid an overshoot in order to prevent an increase in metabolic demands of the spinal cord. As such, on the other end of temperature-related management strategies, systemic cooling or local cooling has been proposed as a measure to minimize oxygen consumption from the spinal cord, thereby reducing potential ischemic damage following ISCI.^46^ While local cooling has not been examined in the context of elective spine surgery, studies in the field of thoracoabdominal aortic aneurysm repair have demonstrated safety and potential neuroprotective effects of cooling the epidural space.^47,48^ Defining specific temperature values for local cooling remains elusive, but the use of 4° iced saline to cool the epidural space and the CSF to approximately 27° has shown promising results in minimizing postoperative neurological complications in the setting of thoracoabdominal vascular surgery.^47^

If possible, any surgeon-related action that may be the underlying cause of an ISCI should be reversed (eg removal/repositioning of misplaced hardware such as screws, interbody cages or bone graft), and any compression on the spinal cord or the nerve roots should be removed (eg osteophytes, hematoma, hemostatic agents). If readily available imaging modalities, such as intraoperative x ray, fluoroscopy or intraoperative ultrasound, do not provide necessary information about potential hardware misplacement or ongoing compression to the spinal cord, appropriate emergent cross-sectional imaging should be considered (such as computed tomography or magnetic resonance imaging). Correction of a deformity is associated with tractive forces on the spinal cord macro- and microvasculature and might therefore result in ischemia-related IONM signal loss.^1^ If such a signal loss is temporally associated with a deformity correction maneuver, correction should be reversed and if signals are not recovered, a moderate correction or a staged procedure may need to be considered. Eventually, if IONM signals do not recover despite the aforementioned management strategies, a Stagnara wake-up test to assess motor function can be performed.

Our results showed that the administration of steroids should be considered in the therapeutic management of an ISCI.^11,17,18,20,21,25-27^ Even though not thoroughly studied in the context of ISCI, the common use of methylprednisolone as an anti-inflammatory/immunomodulatory pharmacotherapeutic likely arises from its extensive study in the field of traumatic SCI.^49-51^ The North American Spinal Cord Injury Study (NASCIS) II therapeutic protocol, whereby a 30 mg/kg bolus of methylprednisolone is given over 15 minutes, followed by a 23 hour infusion of 5.4 mg/kg/hour, was among the most commonly chosen therapeutic schemes.^11,17,18^ The use of methylprednisolone as a neuroprotective drug in the setting of traumatic SCI has been the source of decades-long vibrant debates surrounding its safety and efficacy.^49,50^ And yet, our recent systematic review and meta-analysis, which was conducted as part of the 2017 AO Spine Guideline on the use of methylprednisolone in traumatic SCI, showed modest motor score improvements (3.21 points, [95% CI = .10 – 6.33; *P* = .04]) in patients who received treatment within 8 hours of injury according to the NASCIS II protocol. At the same time, pooled risk of death, wound infection, gastrointestinal hemorrhage, sepsis, pulmonary embolism, urinary tract infection, pneumonia, or decubiti did not show a statistically significant difference between groups.^52^ It should be noted that the NASCIS trials were conducted in patients with closed, non-penetrating SCIs. Patients with ISCI were not studied in the NASCIS trials, and so while the practice of administering high-dose steroids in this circumstance is extrapolated from the situation of non-penetrating traumatic SCI, it should be noted that this is indeed a different clinical context.

## Conclusion

Even though there is a paucity of literature on comparative effects and harms of any of the listed measures, management of intraoperative signal loss and possible SCI merits a standardized checklist and care pathway to avoid and minimize the risk of postoperative neurologic deficits. Such a checklist needs to use simplified language, standardized and non-redundant steps to decrease the risk of human error in a stressful situation.^53^ Dry runs of checklists in high simulation settings will ensure procedural memory and identification of potential pitfalls.^54^ A potential checklist can also be subjected to the “Plan, Do, Study, and Act (PDSA)” cycle to identify shortcomings and improve on them.^55^
